# Salicylic Acid in Acute Rheumatism

**Published:** 1877-03

**Authors:** T. A. Perrin

**Affiliations:** Sheboygan, Mich.


### Salicylic Acid in Acute Rhumatism.

A. McK., aged 35, was the subject of an attack of inflammatory rheumatism, in November, 1875.

November 22d. I was called at ten o'clock in the forenoon, and found my patient in bed, suffering severely and unable to move. Told me this was the third attack in four years. The attack previous to this lasted for eleven weeks. Had been taking gun guaiac, in brandy, for two weeks, but kept getting worse. I put him on the alkaline treatment, with tonics, nourishing diet, etc.; applied chloroform liniment to the swollen and painful joints, and rolled them up in cotton-batting.

November 23d. The joints hot, swollen, and exceedingly painful. Was perspiring freely. Temperature 102°. Pulse 110; tongue thickly coated; bowels constipated; urine high-colored and scanty.

December 10th. Patient greatly depressed in spirits; the joints still swollen, hot and painful; urine scanty; bowels constipated; tongue coated; pulse 100. Having noticed, in the cases which I saw reported, of the use of salicylic acid in this disease, that the relief was almost immediate, I resolved to try it. All previous treatment was discontinued, and I ordered ten grains of salicylic acid, in gelatine capsules, every two hours.

December 11th. Found patient feeling much better; the pain and tenderness was subsiding; bowels had moved once; pulse 90; tongue slightly coated; urine scanty.

December 13. Found patient lying on his side, pain all gone, but the joints felt stiff. Had slept nearly all night; urine not so highly colored; pulse 80; temperature 98°. Ordered the medicine every four hours, for the next twenty-four hours.

December 14th. Patient sitting up in a chair and able to walk very slowly around the room; bowels regular; pulse 80; urine more profuse and not so dark.

December 20th. Patient able to walk several rods out of doors, joints a little stiff, feels week, but gradually recovering strength, appetite good, bowels regular, no pain or tenderness. There has been no return of the disease and no ill effects caused by the medicine. There was no other medicine used while using the silicylic acid, and no local applications. The only disadvantage with the medicine was a considerable irritation of the mouth and throat, and a hot burning sensation in the stomach.

Case 2.—Lizzie K., aged 13, had been suffering for several days with severe pain in both lower extremities. I first saw her at seven o'clock in the evening; ordered three-grain doses of salicylic acid every two hours. Saw her again next day; pain nearly all gone, and feeling quite comfortable. Did not see her again for several days, when I met her on the street. She was up working in the house in four days from commencement of the salicylic acid, and has had no return of the disease.

Case 3.—O. B. W., aged 22, was subject to an attack of inflammatory rheumatism, in March, 1876, affecting the right lower extremity. Ordered him seven grains salicylic acid every two hours. In twenty-four hours the pain and tenderness had all disappeared, but the joints felt stiff. Discontinued the medicine, and in twenty-four hours pain and tenderness in left elbow and wrist. Ordered the salicylic acid as before, and the next day was all right and has had no return of the disease since. After taking two or three doses, patient complained of nausea, but no vomiting, and as soon as the medicine was swallowed, a terrible burning sensation in the stomach. To relieve the burning sensation, I ordered oatmeal gruel, which relieved it almost immediately.—*Med. and Surg. Reporter*.

T. A. Perrin, M. D.

Sheboygan, Mich.

